# FlexED8: the first member of a fast and flexible sample-changer family for macromolecular crystallography

**DOI:** 10.1107/S2059798317013596

**Published:** 2017-09-29

**Authors:** Gergely Papp, Franck Felisaz, Clement Sorez, Marcos Lopez-Marrero, Robert Janocha, Babu Manjasetty, Alexandre Gobbo, Hassan Belrhali, Matthew W. Bowler, Florent Cipriani

**Affiliations:** a European Molecular Biology Laboratory, Grenoble Outstation, 71 Avenue des Martyrs, CS 90181, 38042 Grenoble, France

**Keywords:** sample changer, robotics, automation, dewar, EdgeDewar, SPINE, SPINEplus, miniSPINE, NewPin, gripper, double gripper

## Abstract

Automated sample changers are now standard equipment for modern macromolecular crystallography synchrotron beamlines. Nevertheless, most are only compatible with a single type of sample holder and puck. Recent work aimed at reducing sample-handling efforts and crystal-alignment times at beamlines has resulted in a new generation of compact and precise sample holders for cryocrystallography: miniSPINE and NewPin [see the companion paper by Papp *et al.* (2017, *Acta Cryst.*, D**73**, 829–840)]. With full data collection now possible within seconds at most advanced beamlines, and future fourth-generation synchrotron sources promising to extract data in a few tens of milliseconds, the time taken to mount and centre a sample is rate-limiting. In this context, a versatile and fast sample changer, FlexED8, has been developed that is compatible with the highly successful SPINE sample holder and with the miniSPINE and NewPin sample holders. Based on a six-axis industrial robot, FlexED8 is equipped with a tool changer and includes a novel open sample-storage dewar with a built-in ice-filtering system. With seven versatile puck slots, it can hold up to 112 SPINE sample holders in uni-pucks, or 252 miniSPINE or NewPin sample holders, with 36 samples per puck. Additionally, a double gripper, compatible with the SPINE sample holders and uni-pucks, allows a reduction in the sample-exchange time from 40 s, the typical time with a standard single gripper, to less than 5 s. Computer vision-based sample-transfer monitoring, sophisticated error handling and automatic error-recovery procedures ensure high reliability. The FlexED8 sample changer has been successfully tested under real conditions on a beamline.

## Abstract 2

A sample changer based on a six-axis industrial robot and a new sample-storage dewar with an ice-cleaning feature have been developed to open automated X-ray crystallography beamlines to new sample-holder models, such as miniSPINE and NewPin, while remaining compatible with the widespread SPINE sample-holder standard.

## 1. Introduction   

Following the pioneering developments in automating sample transfer at macromolecular crystallography (MX) beamlines, and the standardization of sample holders used in cryo­crystallography at the turn of the century (Cipriani *et al.*, 2006 ▸; Cohen *et al.*, 2002 ▸; Jacquamet *et al.*, 2004 ▸, 2009 ▸; Ohana *et al.*, 2004 ▸; Pohl *et al.*, 2004 ▸), sample changers continue to develop to match the ever-increasing demand for storage capacity, transfer speed and fully automated data collection (Nurizzo *et al.*, 2016 ▸; Russi *et al.*, 2016 ▸; Ferrer *et al.*, 2013 ▸). Currently, 15 distinct types of sample changer, six types of basket and six types of sample holder are deployed at the various MX beamlines around the world (http://smb.slac.stanford.edu/robosync/). Some of the existing sample changers are equipped with tool changers that are able to switch between standard data collection and *in situ* data collection (Jacquamet *et al.*, 2004 ▸), but most are only compatible with a single sample holder and puck model currently used in cryocrystallography.

**Figure 1:**
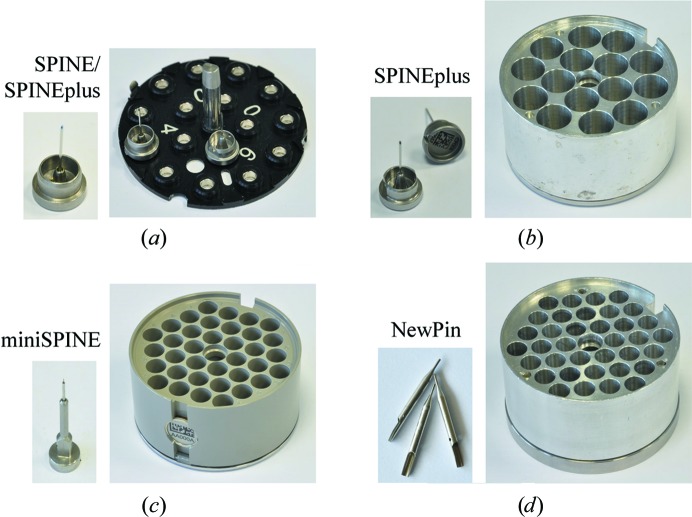
Sample holders and pucks compatible with FlexED8. (*a*) SPINE and SPINEplus pins (cap diameter 12 mm) and uni-pucks. (*b*) SPINEplus pins (cap diameter 12 mm) and puck. (*c*) MiniSPINE pin (cap diameter 7 mm) and puck. (*d*) NewPin (diameter 1.9 mm) pins and puck. (All of the pucks have the same footprint with a diameter of 67 mm.)

Here, we describe a flexible automated sample changer based on a six-axis industrial robot, cryo-grippers, sample-storage dewar and control system. The operation and alignment procedures for use of the sample changer are also described as well as its deployment at synchrotron beamlines. The Flex robotics can handle different sample-holder models stored in uni-puck footprint-style pucks. It is compatible with sample holders compliant with the SPINE standard (http://instrumentation.embl.fr/spinesampleholder) and can handle new sample holders designed to minimize sample-handling effort and reduce crystal-alignment time at MX beamlines: SPINEplus, miniSPINE and NewPin (Fig. 1 ▸; Papp *et al.*, 2017 ▸). An integrated tool changer allows the use of different robot grippers and opens the device to future sample-holder models.

**Figure 2:**
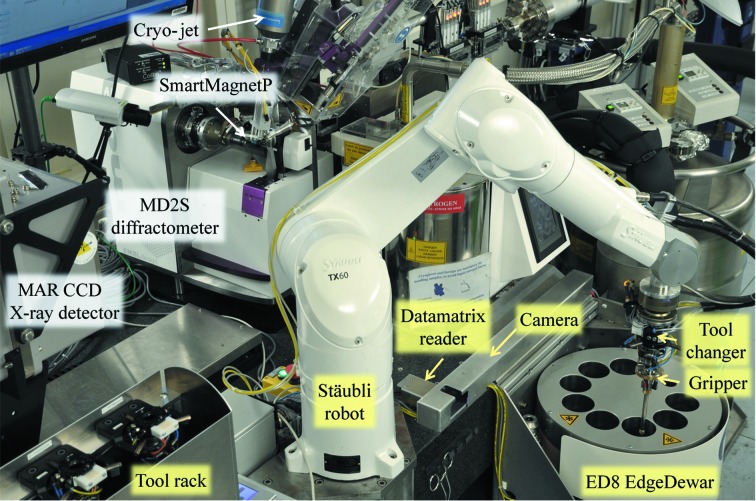
The FlexED8 sample changer shown at ESRF beamline BM14. MD2S diffractometer with SmartMagnetP, cryo-jet and MAR CCD X-ray detector, Flex robotics with a tool rack, Stäubli TX 60L six-axis robot equipped with a tool changer and a miniSPINE/NewPin gripper, Datamatrix reader, camera and ED8 EdgeDewar.

This paper focuses on the FlexED8 sample-changer model (Fig. 2 ▸), the first member of a family of sample-handling devices based on the Flex robotics that can be adapted to different dewars and experimental environments but retains the same core robotics and control software. FlexED8 includes an eight-puck storage dewar, the EdgeDewar (ED8), in which sample pucks with a uni-puck-style footprint are stored in an open vessel that is continuously filled with ice-filtered liquid nitrogen. This patented design facilitates access to the samples while minimizing ice contamination. FlexED8 has been tested under real conditions with SPINE, SPINEplus, miniSPINE and NewPin sample holders on the EMBL–ESRF–India BM14 CRG beamline (Papp *et al.*, 2017 ▸). The second member of the Flex family, the FlexHCD (Supplementary Fig. S7), was developed in collaboration with the ESRF. This sample changer uses a modified version of the ESRF High Capacity Dewar (HCD; Bowler *et al.*, 2015 ▸; Nurizzo *et al.*, 2016 ▸) instead of the EdgeDewar. It can simultaneously hold up to 12 SC3 pucks (Cipriani *et al.*, 2006 ▸) and 12 pucks with a uni-puck-style footprint. In user operation since January 2016 on ESRF beamline ID30B (Mueller-Dieckmann *et al.*, 2015 ▸), the FlexHCD sample changer can transfer SPINE sample holders with or without vials using a flipping gripper commercialized by Irelec-Alcen, Saint-Martin-d’Hères, France or an in-house-developed uni-puck-compliant gripper, respectively. It is also compatible with miniSPINE and NewPin sample holders, and is being equipped with a gripper to transfer SBS crystallization plates for *in situ* data collection (Supplementary Fig. S6). The third member of the Flex family is the FlexED3 (Supplementary Fig. S7), a sample-recovery and cryo-storage system that includes a three-slot EdgeDewar (ED3). Associated with a CrystalDirect harvester (Cipriani *et al.*, 2012 ▸; Zander *et al.*, 2016 ▸) it can automatically recover up to 30 SPINE sample holders with harvested crystals in cryo-vials and store them in three SC3 pucks. The flexibility of these robotics already provides automation at many stages of MX experiments and can adapt seamlessly to their rapidly changing sample environments.

## 2. Experimental details   

### 2.1. The Flex sample-transfer robotics   

The Flex robotics (Fig. 2 ▸) are built around a TX60L six-axis industrial robot (Stäubli Faverges SCA, Faverges, France) powered by a CS8C controller. Ancillary functions are controlled through Modbus electronic modules (WAGO Contact SAS, Roissy, France) connected to the CS8C *via* an ethernet port (Supplementary Fig. S1). In addition to the robot arm, the Flex robot includes the following (Fig. 2 ▸): (i) a tool changer mounted on the robot arm and a tool-storage rack that can hold different grippers and an alignment probe; (ii) a machine-vision system used to calibrate the grippers and control the position of the sample holders in the gripper during their transfer; (iii) a Datamatrix reader to track the samples and (iv) a deicing station to remove frost accumulated on the grippers during sample manipulation.

The precision of the robot is a key parameter to successfully handle small sample holders in a high-density storage environment. If the positioning repeatability of compact six-axis industrial robots is typically a few tens of micrometres at the mounting interface of axis 6 (±30 µm for the Stäubli TX60L robot), the positioning repeatability at the tip of a gripper depends on its length and can exceed several hundred micrometres. Moreover, the absolute positioning precision of industrial six-axis robots is much worse than their positioning repeatability. The mean absolute positioning error at the tool-mounting interface of a TX60L Stäubli robot arm measured by the manufacturer over 358 points within the whole working area of the robot is ±0.54 mm (manufacturer’s documentation; Stäubli Faverges SCA, Faverges, France). In addition, the angular error of the tool-mounting interface is not specified; hence, the worst absolute positioning error at gripper tips could be amplified by the length of the grippers.

**Figure 3:**
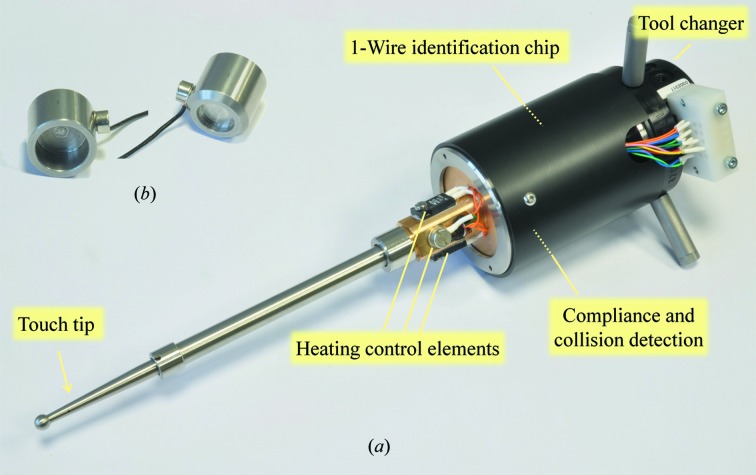
(*a*) Touch probe with tool-changer interface, compliance- and collision-detection system, 1-Wire identification chip (under the cover, similar to the gripper base), heating-control elements and touch tip. (*b*) Goniometer-alignment tool shown from the palpating side (left) and goniometer-mount side (right) with grounding electrical wire.

Nevertheless, in our case, the observed relative positioning error at gripper tips over a restricted working area, such as a puck, is estimated to be less than ±0.2 mm. Therefore, to minimize the gripper-positioning error over the pucks, we created a local coordinate system for each puck in the dewar. Similar to the calibration procedure of the SAM (Smith & Cohen, 2008 ▸) sample-changer system, the absolute position of each local coordinate system is learned using a touch probe (Fig. 3 ▸ *a*) and the robot movements inside the coordinate system are executed relative to the reference positions. Consequently, the overall positioning error of the grippers against the sample holders in a puck remains smaller than ±0.3 mm. This is sufficient to reliably handle the NewPin and miniSPINE sample holders. Position-learning procedures, using different alignment tools and a machine-vision system, have been implemented to calibrate the geometry of the robot grippers and define the positions of the dewar puck slots and goniometer mount in three dimensions. In order to prevent misalignment caused by thermal dilation, the calibration procedures are executed under cryoconditions. Finally, for security during transfers, the grippers have a built-in compliance- and collision-detection system. Additionally, the dewar slots are equipped with in-house-developed precise puck presence and type detection sensors (ProxiSense) to ensure that the pucks are well positioned in the dewar slots and to prevent collisions owing to gripper–puck mismatch or pucks being unexpectedly removed by grippers during sample transfer. (The ProxiSense system is commercialized as part of Flex robotics by Irelec-Alcen, Saint-Martin-d’Hères, France.)

### 2.2. The cryo-grippers   

**Figure 4:**
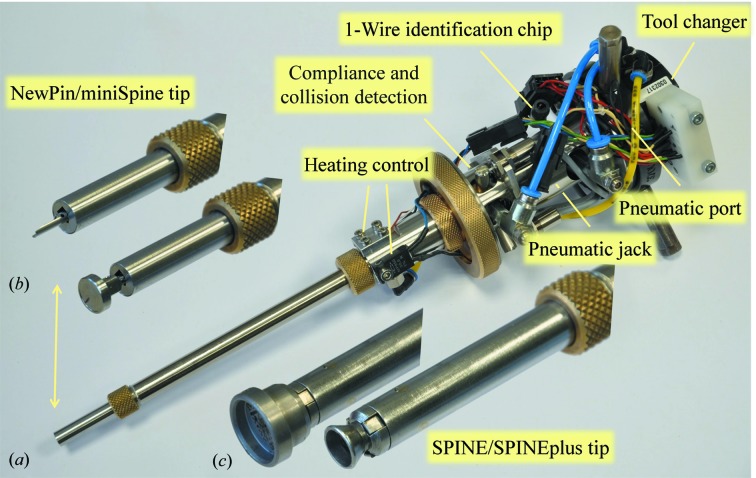
Cryo-grippers. (*a*) Gripper base equipped with a NewPin/miniSPINE tip that includes a tool-changer interface, a 1-Wire identification chip, a pneumatic jack inside the body, a compliance- and collision-detection system, heating-control elements and a pneumatic port for inner deicing of the gripper. (*b*) NewPin/miniSPINE tips holding a NewPin and a miniSPINE Pin. (*c*) SPINE/SPINEplus tip empty and holding a SPINE pin.

Transferring cryocooled crystals without using vials filled with liquid nitrogen requires a gripper that maintains the crystals at cryogenic temperature while protecting them from ice formation during their transfer in air. Two different grippers that share a common base have been developed (Fig. 4 ▸). The first is dedicated to the NewPin and miniSPINE sample holders (Fig. 4 ▸ *b*) and the second to the SPINE/SPINEplus sample holders (Fig. 4 ▸ *c*). The common base includes (i) a tool-changer interface; (ii) a compliant collision-detection system connected to a fast input of the CS8C robot controller; (iii) a write-protected EEPROM chip using a 1-Wire communication protocol for gripper identification; (iv) a pneumatic jack to actuate the sample-holder locking mechanism of the gripper; (v) a heating resistor and a thermocouple to keep the base of the gripper at room temperature when its tip is parked in liquid nitrogen and (vi) a pneumatic port for deicing the inner part of the gripper tips with dry air or nitrogen gas. Both grippers hold the crystal mount of the sample holders within a small cavity, protecting the crystals from icing and maintaining them at cryogenic temperature during transfer. Before transferring a sample, the tip of the gripper is cooled in liquid nitrogen. In order to increase the availability of the sample changer, the gripper remains parked in liquid nitrogen between transfers. It is automatically deiced every 16 transfer cycles. To reduce the deicing time, a flow of compressed air is applied outside the gripper tip while nitrogen gas or dry air at room temperature is blown inside the gripper tube. The de-icing process takes approximately 60 s, immediately followed by the cooling process consisting of the immersion of the gripper in liquid nitrogen. The entire deicing and cooling procedure takes 1 min 30 secs.

The NewPin/miniSPINE gripper grabs the sample holders on their 1.9 mm diameter pin section (Papp *et al.*, 2017 ▸; Fig. 4 ▸ *b*). The pins are blocked in the V-shaped groove of the tip of the gripper by a blade moved by the pneumatic actuator of the gripper base. The material and geometry of the NewPin/miniSPINE gripper tip allows an RFID tag to be read from outside the gripper while they are transferred. The SPINE/SPINEplus gripper grabs the caps from their internal 8 mm diameter groove (Fig. 4 ▸ *c*). The caps are locked in the tip of the gripper by three blades set at 120° that slip on a cone and rest against the 8 mm inner diameter of the caps. The three blades are actuated by the pneumatic jack of the gripper base. The use of a tool changer in combination with gripper IDs, puck IDs and the puck-type detection system allows safe transfer between different sample holder models and minimizes the risk of collision owing to gripper and puck mismatch.

### 2.3. The sample-storage dewar EdgeDewar ED8   

**Figure 5:**
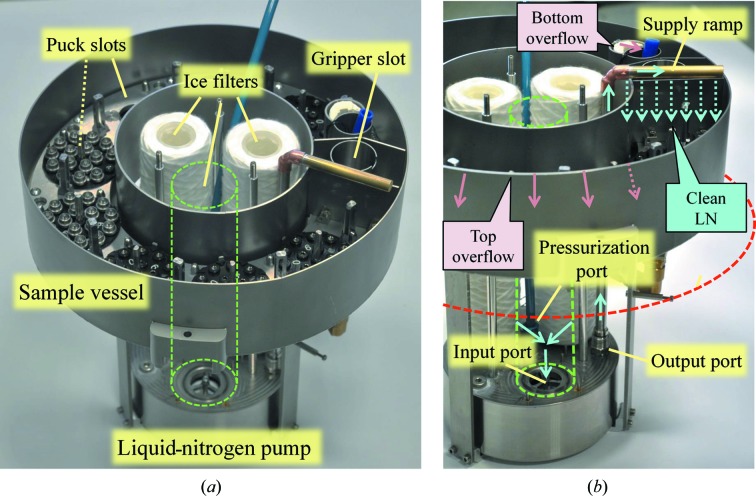
EdgeDewar ED8. (*a*) Sample vessel with eight puck slots, the gripper parking slot and the liquid-nitrogen pump with three ice filters at the top (one is removed for clarity and represented as a dashed green line). (*b*) Detail of the ice-filtering system showing the liquid-nitrogen pump with one of its three filling ports connected to an ice filter (removed) and equipped with a closing valve connected to a float inside the pump, the pressurization pipe and the output pipe connected to the supply ramp. The red dashed line indicates the average level of liquid nitrogen in the main dewar vessel.

The EdgeDewar ED8 is composed of a 40 cm diameter vacuum-insulated dewar with a separate sample vessel that holds the pucks (Fig. 5 ▸, Supplementary Fig. S8). This sample vessel is continuously filled with ice-filtered liquid nitrogen pumped from the main dewar vessel that acts as a reservoir. Any ice floating in the sample vessel flows out from the top edges in the same manner as dirt in an edge (swimming) pool and the sinking ice is drained from the bottom. The ice-contaminated liquid nitrogen returns to the main dewar to be filtered and re-injected into the sample vessel. Several advantages result from this design. (i) The quantity of ice in the liquid nitrogen of the sample vessel, and therefore sample icing, is reduced. This is an important feature when a large number of samples are stored without vial protection (such as uni-pucks, miniSPINE and NewPin pucks). (ii) The top of the dewar can stay open as water contamination from humidity in the ambient air is continuously removed. (iii) The level of liquid nitrogen above the samples is constant and can be minimized to facilitate access to the pucks from the top of the dewar, resulting in shorter grippers and increased positioning precision of the robotics. (iv) The level of liquid nitrogen in the main dewar is not critical and can accommodate automated filling systems with large-level hysteresis. Finally, (v) the level of liquid nitrogen in the main dewar can be relatively low, thus increasing the thermal bridge between the surface of liquid nitrogen and the top of the dewar, potentially leading to a reduction in the consumption of liquid nitrogen. The level of the liquid nitrogen is controlled by digital sensors and is maintained between 5 and 15 cm above the base of the ice filters (string-wound polypropylene 1 µm filter cartridge). The liquid-nitrogen pump sits below the sample vessel, fully immersed in liquid nitrogen. The only moving part of the pump is the valve of the input port situated at the top of the chamber and connected to a float set underneath, inside the chamber of the pump. The operation of the pump is controlled by cyclically injecting pressurized nitrogen gas in the chamber of the pump though a pressuring port. When empty, the chamber of the pump fills by gravity, with ice-filtered liquid nitrogen flowing through the input port, until the valve closes by being pushed by the float. When the chamber is connected to the nitrogen-gas supply the overpressure pushes the liquid nitrogen out of the chamber though an output port situated at the bottom of the chamber and connected to a pipe that ends above the sample vessel. At the same time, overpressure locks the input valve, keeping the input port closed, until the chamber is empty and/or the gas supply is stopped. The float then falls down, reopening the input port. The pressure of nitrogen gas injected, typically 50 kPa, and the off/on times, typically 20 s off and 5 s on, are set such that the chamber has time to fully fill and empty with minimum dead times and gentle liquid-nitrogen flow in the sample vessel. Digital sensors monitor the level of liquid nitrogen in the sample vessel to ensure that it is continuously filled. The dewar is automatically filled from the liquid-nitrogen network of the beamline. Owing to the poor insulation of the liquid-nitrogen network, a phase separator was placed next to the dewar to limit the flow of nitrogen gas in the buffer vessel at the beginning of refilling phases. Depending on the turnover of pucks, the EdgeDewar ED8 and ice filters need to be deiced once every one to two weeks. The process to evaporate the liquid nitrogen, and the water coming from ice accumulated over time, including the water trapped in the ice filters, takes about 6–8 h. The rate of drying could be further increased by mounting a cover over the dewar equipped with a draining pipe and by putting the dewar under pressure such that the liquid nitrogen and water is quickly drained out before drying.

**Figure 6:**
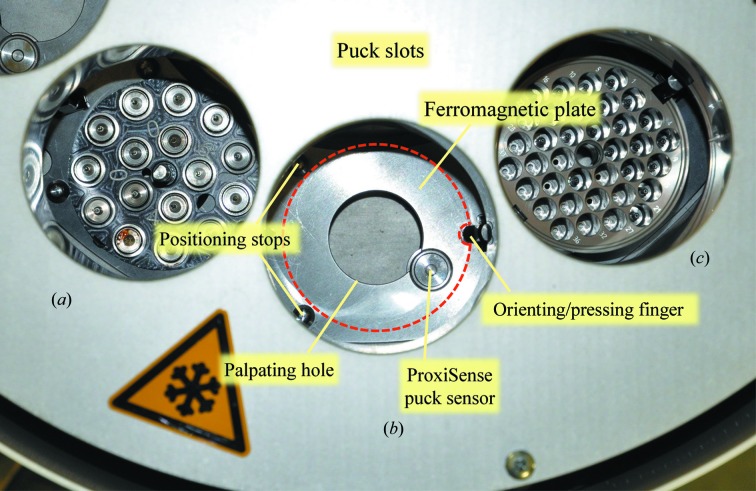
Puck slots. (*a*) With a uni-puck inserted. (*b*) Empty, showing the ferromagnetic plate, the two radial positioning stops, the orienting and pressing finger, the ProxiSense puck sensor and the alignment gripper-palpating hole. (*c*) With a miniSPINE puck inserted.

The eight puck slots are accessible from eight openings on the top of the EdgeDewar and can receive pucks with uni-puck-style footprint. The pucks sit on a ferromagnetic plate, where they are held in place by the attraction of their own magnets to the plate. The positioning/guiding fingers that are present on some existing uni-puck Dewar slot models, such as the CATS sample changer (http://www.irelec-alcen.com/fr/produits/robotique), were not retained as they would have led to a reduction in the number of sample holders stored in miniSPINE and NewPin pucks. Furthermore, an improved accuracy in puck positioning was required for reliable robotic handling of miniSPINE and NewPin sample holders. To ensure a precise and play-free positioning of the pucks, they are positioned from the outside by two cylindrical stops on which they are placed by an orienting and pushing finger that fits in the notches of their bases (Fig. 6 ▸). A puck-detection system, ProxiSense (Supplementary Fig. S1), based on an analogue magnetic proximity sensor, ensures the reliable detection of the presence and positioning of the pucks in the dewar slots. The analysis of the ferromagnetic signature of the base of the puck measured at different frequencies also allows recognition of the puck type installed. The presence of the pucks can be checked at any time by sending RS232 queries to the ProxiSense control card. The card also provides a hardware signal to monitor, in real time, the correct positioning of the puck selected for sample transfer. This allows the robot to immediately stop if the puck is accidentally removed from the dewar by the gripper.

### 2.4. Control of the FlexED8 sample changer   

The sample changer is controlled by high-level software written in Java, based on the JLib library (EMBLEM Technology Transfer GmbH, Heidelberg, Germany; http://software.embl-em.de). The control software runs on a Windows PC, accessible by a graphical user interface (Supplementary Figs. S3 and S4), and a dynamically generated socket server on the local network using the Exporter protocol (part of the JLib library). The *MXCuBE* experiment-control software (Gabadinho *et al.*, 2010 ▸) connects to this server, providing user access to the basic functionalities and error messages (Supplementary Fig. S5). Alternative communication protocols such as Tango, Tine, Epics or Java Web Services are also available for integration in other beamline-control environments. Besides the exported basic functions, the Java software allows (i) the display of a contextual GUI for the different working modes (puck loading or sample transfer), (ii) monitoring of critical parameters of the system, such as the gripper temperature or the dewar liquid-nitrogen level, (iii) the display and storage of log messages and activity results, (iv) the execution of image-processing tasks, (v) communication with peripheral devices (such as Datamatrix readers, ProxiSense control card, industrial cameras, 1-Wire IDs) and (vi) communication with other beamline devices (for example the gonio­meter). The movements of the robot arm and the time-critical processes (low-level machine control) are coded in the VAL3 programming language (Stäubli Faverges SCA, Faverges, France; https://www.staubli.com/en/robotics/robot-software/val3-robot-programming/val-3-language/) and are executed in the CS8C robot controller. The communication between the high-level control and the low-level robotic control relies on the StaubCom library (EMBLEM Technology Transfer GmbH, Heidelberg, Germany; http://software.embl-em.de) that exposes a generic VAL3 socket server on the network to provide a task-oriented programming environment. This low-level VAL3 software (i) dynamically loads and executes movement sequences, (ii) sends feedback messages to the high-level Java software, (iii) executes regulation loops, (iv) exposes IO states and commands to the Java software, (v) notifies the high-level control software of upcoming errors, warnings and log messages during the execution of low-level tasks and (vi) takes actions in case of errors and failures to preserve the integrity of the sample and maintain the operability of the machine for further tasks.

### 2.5. Beamline setup   

The FlexED8 sample changer is associated with an MD2S diffractometer (ARINAX, Moirans, France; http://www.arinax.com) equipped with an MK3 mini-Kappa gonio­meter head (Brockhauser *et al.*, 2013 ▸) and a MAR CCD 225 (marXperts GmbH, Norderstedt, Germany) X-ray detector (Fig. 2 ▸); all of which are fully integrated into the *MXCuBE* beamline-control GUI on BM14 at the ESRF. Two different goniometer mounts were used on the diffractometer to process SPINE/SPINEplus, miniSPINE and NewPin sample holders. The newly developed Parallel Pole SmartMagnet (SmartMagnetP) goniometer mount (Papp *et al.*, 2017 ▸) was used to hold and detect the presence of the SPINE/SPINEplus and miniSPINE sample holders. This modified version of the SmartMagnet (Cipriani *et al.*, 2006 ▸) was integrated into the electronics of the MD2S diffractometer. The software of the standard SmartMagnet controller card was upgraded to make it compatible with the SmartMagnetP. The magnetization command of the SmartMagnetP and the pin-presence detection signals were hardware-connected to the FlexED8 control electronics. Two different detection thresholds are used to reliably detect the presence of SPINE/SPINEplus and miniSPINE sample holders. The detection thresholds are changed manually using the control software of the MD2S, but can be further automated to make simultaneous use of miniSPINE and SPINE/SPINEplus sample holders possible. The NewPin goniometer mount was used to hold NewPin sample holders on the goniometer. No sample-holder detection was provided in this prototype goniometer mount.

### 2.6. Alignment procedures   

The alignment of the robot arm with the goniometer mount and dewar puck slots, as well as the calibration of robot grippers, can be performed automatically. The alignment process is based on the detection of an electrical contact between a target object and the spherical palpating tip of the touch probe (Fig. 3 ▸ *a*). In the dewar, the touch probe palpates the precisely machined alignment holes present in the centre of each puck slot (Fig. 6 ▸ *b*) and the surface of the plate next to the holes. The alignment points are stored and used to match the theoretical spatial layout of the samples with the real dewar slot positions. For alignment with the goniometer, a precisely machined alignment cap (Fig. 3 ▸ *b*) is manually mounted on the goniometer head. The robot then palpates this cap with the touch probe to acquire the three-dimensional position of the goniometer mount. Once the dewar and goniometer alignment procedures have been executed, the robot proceeds to the calibration of the different grippers. This procedure uses a machine-vision system to determine the *x*, *y* and *z* offsets of the tip of the grippers against the tip of the touch probe.

### 2.7. Sample-transfer procedures   

All robot-movement sequences are written in the VAL3 programming language (PLC-IEC 61131-3 compliant) and are executed by the CS8C Stäubli robot controller running a VxWorks real-time operating system that ensures robust and reliable code execution. Various checks are performed during sample-loading and sample-unloading processes at different points of the gripper trajectory. If an abnormal situation is detected, decisions are automatically taken to preserve the integrity of the samples and keep the device available for further actions. A sample-recovery puck is installed in position 8 of the ED8 to store samples that cannot be mounted on the goniometer or replaced in their original puck. The robot grippers are parked in liquid nitrogen between successive sample-loading and sample-unloading cycles, and are deiced after 16 sample transfers. In a typical loading or unloading procedure, the system performs the following checks: (i) when the gripper is in the vicinity of a puck, the system monitors the gripper collision-detection system and the ProxiSense puck-presence signal to detect any potential collision or removal of the puck from the dewar, (ii) during both loading and unloading procedures a pin-detection station placed in the middle of the robot trajectory and equipped with a machine-vision system checks whether the pin is well positioned in the gripper and a Datamatrix reader simultaneously registers the sample-holder Datamatrix code (if any) and (iii) when removing or placing a sample on the goniometer the system monitors the gripper collision-detection signal and ensures that the signals ‘cryo is back’, ‘transfer is authorized’ and ‘sample is present’ provided by the goniometer are in the proper state to prevent any collision with the goniometer, sample holder or cryo-jet nozzle.

## 3. Results   

The most important aspect of a robotic sample changer is its ability to preserve the integrity of the crystals during transfer between the storage dewar and goniometer. The temperature at the position of the crystal should be maintained below 100 K and ice contamination avoided during transfer. These points are governed by the design of sample holders, the associated robot grippers and by the transfer speed. The temperature at the sample position in the grippers during the load and unload transfer processes was therefore measured, and the limits determined at which the diffraction quality starts to degrade against the transfer time and against repeated transfers of the same crystal. The repositioning precision of the different sample holders on the goniometer was also measured as it directly affects the time required to align crystals in the X-ray beam. All tests were carried out on the EMBL–ESRF–India BM14 beamline, with the ED8 filled with liquid nitrogen and the temperature of the cryo-jet set to 100 K. The tip of the cryo-jet head was adjusted to a distance of 5 mm from the nominal position of the crystals and a 3 mm retraction was systematically applied during the gripper approach to avoid collision, although this is only necessary when transferring SPINE/SPINEplus sample holders.

### 3.1. Temperature inside the grippers at the crystal position during the transfers   

The temperature inside the grippers at the crystal position during loading–unloading procedures was measured with a thermocouple (Thermocouple RS Pro Type T). Three loading–unloading cycles were executed at a nominal transfer speed with each gripper. Additionally, to estimate the maximum loading time at which the temperature at the crystal position remained below 100 K, the robot speed was reduced by a factor of ten and the temperature at the sample position was recorded over time with each gripper.

**Figure 7:**
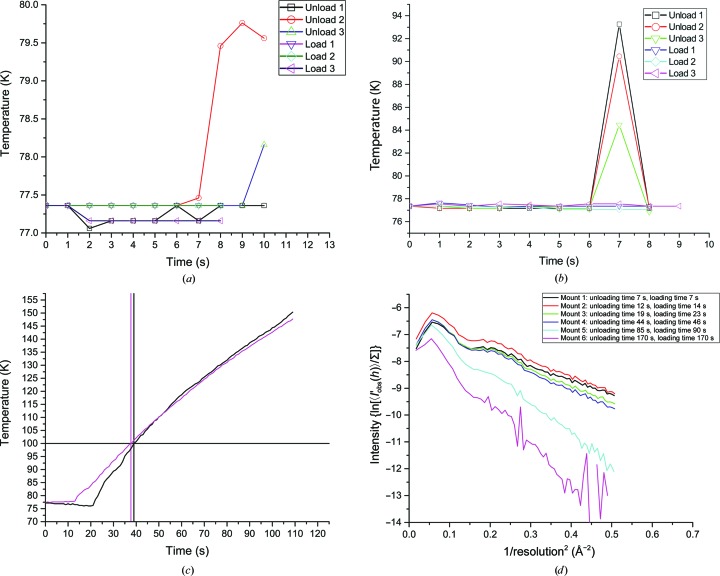

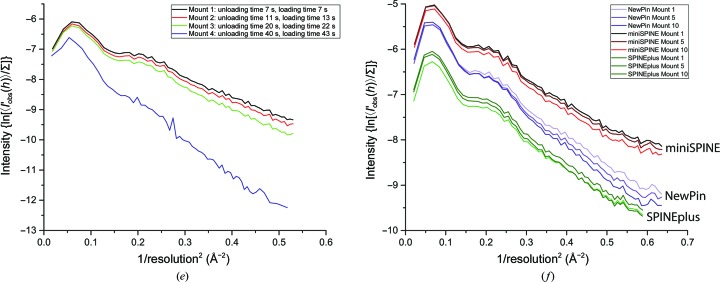
Evolution of temperature within grippers and crystal integrity during transfer. (*a*) Measured temperature in the miniSPINE/NewPin gripper during successive loading and unloading at nominal robot speed. (*b*) Measured temperature in the SPINEplus gripper during successive loading and unloading at nominal robot speed. (*c*) Temperature evolution in the miniSPINE/NewPin gripper (magenta) and SPINEplus gripper (black) during low-speed loading. The horizontal line marks the 100 K level and the vertical lines show the time at which this level is reached for each gripper. (*d*) Wilson plots from data collected during successive mounts with increasing loading/unloading times for the same sample in the miniSPINE/NewPin gripper. (*e*) Wilson plots from data collected during successive mounts with increasing loading/unloading times for the same sample in the SPINEplus gripper. (*f*) Wilson plots from data collected from thaumatin crystals after multiple mounting and unmounting cycles at nominal speed with NewPin, miniSPINE and SPINEplus sample holders.

During standard loading procedures the temperature inside the miniSPINE and SPINEplus grippers remained below 78 K, and it remained below 80 and 93 K during standard unloading procedures, respectively (Figs. 7 ▸ *a* and 7 ▸ *b*). Higher temperature maxima are observed during the unloading procedures as the grippers remain out of liquid nitrogen on their way from the dewar to the goniometer and the unloading procedure begins with a gripper that has started to warm up. During the unloading procedures, the temperature in the SPINEplus gripper starts increasing after 6 s, the time at which liquid nitrogen trapped inside the gripper is fully evaporated, rises to 93 K and decreases to 77 K after 7 s, the time at which the gripper returns back to the dewar and is filled by liquid nitrogen (Fig. 7 ▸ *b*). A similar phenomenon can be observed with the miniSPINE gripper but with a smaller temperature increase, because its pin-locking system holds liquid nitrogen more efficiently inside the gripper tip when the gripper is in the closed position without a pin (Fig. 7 ▸ *a*). However, at the beginning of loading procedures the grippers are plunged into liquid nitrogen when taking samples from the dewar; therefore, this procedure starts with a gripper at 77.4 K with some liquid nitrogen trapped in the grippers. It can therefore be concluded that the grippers act as an efficient cold buffer and maintain the samples below 100 K during standard transfer procedures. The temperature at the sample position in the grippers was recorded during low-speed loading procedures to determine the times at which the sample reaches 100 K (Fig. 7 ▸ *c*). These limits are 38 and 39 s for the miniSPINE and SPINEplus grippers, respectively.

### 3.2. Tests with model protein   

Thaumatin was selected as a test protein. Crystals were grown by the hanging-drop method using a protein concentration of 15 mg ml^−1^ in a solution consisting of 100 m*M* *N*-(2-acetamido)-2-iminodiacetic acid (ADA) pH 6.5. The reservoir solution consisted of 100 m*M* ADA pH 6.5 and 0.9–1.0 *M* sodium/potassium tartrate. Crystallization droplets consisting of 2 µl protein solution and 2 µl reservoir solution were equilibrated against 1 ml reservoir solution at room temperature. The crystals were manually harvested with the different types of sample holders and were briefly immersed (∼2 s) in cryobuffer consisting of 0.75 *M* sodium/potassium tartrate and 25%(*v*/*v*) glycerol before being cooled directly on the MD2S goniometer at 100 K. The diffraction data sets were collected at an energy of 13 keV with the long *c* unit-cell dimension of each crystal oriented along the spindle axis to avoid overlaps of reflections on the detector (Dauter, 1999 ▸). For NewPin sample holders, the same starting crystal orientation was easily achieved for each repeated data collection by using the automatic reorientation system of the NewPin goniometer mount (Papp *et al.*, 2017 ▸). For the miniSPINE and SPINEplus sample holders, the initial crystal orientation was manually corrected using the MD2S goniometer. In order to expose the same crystal volume, data sets of 240 images with 0.5° oscillation range were collected. The exposure time per frame was selected to ensure that no overloaded spots were recorded in test images taken 90° apart. The dose for data sets was estimated using *RADDOSE*-3*D* (Zeldin *et al.* 2013 ▸) at 0.42 MGy. This dose is well below the ‘Garman limit’ of 30 MGy (Owen *et al.* 2006 ▸), meaning that significant radiation damage should not occur in the tests performed here. All data sets were processed using *HKL*-2000 and, for consistency, the resolution of the data sets was truncated where the empirical signal-to-noise ratio, 〈*I*/σ(*I*)〉, dropped below 2.0 in the highest resolution shell.

#### 3.2.1. Crystal preservation *versus* transfer time   

The main objective of these measurements was to ensure that at nominal robot transfer speeds the grippers protect the crystals from thermal deterioration and icing with a sufficient safety margin. This was evaluated by transferring test crystals at robot speeds decreased from the nominal value to the point where the diffraction quality started to degrade. The NewPin/miniSPINE gripper was tested first, using a miniSPINE sample holder. The robot was initially set at its nominal speed, with a 7 s sample-unloading/loading time. A thaumatin crystal was manually mounted on the goniometer of the MD2S diffractometer, cooled in the cryostream and subsequently unloaded and loaded by the robot at nominal speed. Five data sets were then collected successively. After each collection, the crystal was unloaded to the EdgeDewar and remounted onto the gonio­meter with a transfer time incrementally doubled for every successive transfer. The Wilson plots were then compared for each data set to show any changes in crystal quality and the appearance of crystalline ice (Fig. 7 ▸ *d*). After transfer at nominal speed and a second transfer with a 19/23 s unloading/loading time, the Wilson plots did not show any significant deviation. After a transfer with 44/46 s loading/unloading times the Wilson plot clearly indicates that the crystal was losing its diffraction power as the intensity drops significantly. After a transfer with 85/90 s unloading/loading times the resolution dropped to 1.8 Å. Crystalline ice rings also appeared on the diffraction images in the corresponding regions (for example at ∼3.7 Å resolution), confirming the ice formation visible on the sample. Finally, after a transfer with 170 s loading/unloading time and five data sets collected in total, the diffraction power of the crystal was lost.

A similar experiment was performed with the SPINEplus gripper and a SPINE pin (Fig. 7 ▸ *e*). The robot was initially set at its nominal speed, with a 7 s sample-unloading/loading time. The first data set was collected at 1.37 Å resolution and four data sets were successively collected. No significant deviation in the Wilson plot was observed after 11/13 s and 20/22 s unloading/loading-time transfers. After a transfer with 40/43 s unloading/loading times, the Wilson plot clearly shows a significant decrease in intensity and useful data were only obtained to 1.8 Å resolution. No ice rings were observed in the diffraction pattern in the first three data sets. However, ice formation was clearly visible on the sample in the fourth data collection, and ice rings were observed on the diffraction images at the corresponding regions (for example at ∼3.7 Å resolution). These experiments show that for both grippers used at nominal speed the integrity of crystals is preserved with a comfortable safety margin.

#### 3.2.2. Crystal preservation during successive transfers   

The preservation of a crystal during multiple transfers is important as crystals are often screened for diffraction quality before a complete data set is collected from those displaying the best diffraction characteristics (Bowler *et al.*, 2010 ▸). The effect of successive crystal transfers was evaluated for each sample-holder type with its corresponding gripper at nominal robot speed, using the following experimental protocol. A thaumatin crystal manually mounted on the goniometer and flash-cooled in the cryostream was cyclically unloaded/loaded to and from the EdgeDewar. Complete data sets were collected after the first, fifth and tenth transfer cycles. This experiment was repeated twice with each sample-holder model to ensure consistency of the results. For the first trial, the data sets were collected at 1.25 Å resolution for the miniSPINE sample holders, 1.34 Å resolution for NewPin sample holders and 1.35 Å resolution for SPINEplus sample holders. For the second trial, the data sets were collected at 1.25 Å resolution for the miniSPINE sample holders, 1.25 Å resolution for NewPin sample holders and 1.35 Å resolution for the SPINEplus sample holder. None of the samples displayed any deterioration in the diffraction limit, even after ten successive mount/unmount cycles. The Wilson plots are shown for the first trial in Fig. 7 ▸(*f*), indicating that there is no change in the data quality after repeated mounting cycles. No ice formation was observed in any data set collected.

### 3.3. Tests with Axe2   

The miniSPINE sample holder is proposed as a near-future standard; therefore, supplementary tests were performed with samples provided by a user, which may not be as robust as thaumatin. Crystals of Axe2, an intracellular acetylxylooligosaccharide esterase from *Geobacillus stearothermo­philus*, were used for this experiment. A similar data-collection procedure to that used for the thaumatin samples was adopted. Complete data sets were collected after the first, fifth and tenth transfer cycles at 2.5 Å resolution in the first trial and 3.3 Å resolution in the second trial. For each data set, a total of 120 images were collected with a 1° oscillation range. Exposure times of 5 and 4 s per image were used for the first and second trials, respectively. All data sets were processed using *HKL*-2000. As with the thaumatin crystals, no significant changes in the Wilson plots were observed between mounting cycles.

### 3.4. Repositioning precision of the sample holders on the host goniometer   

**Figure 8:**
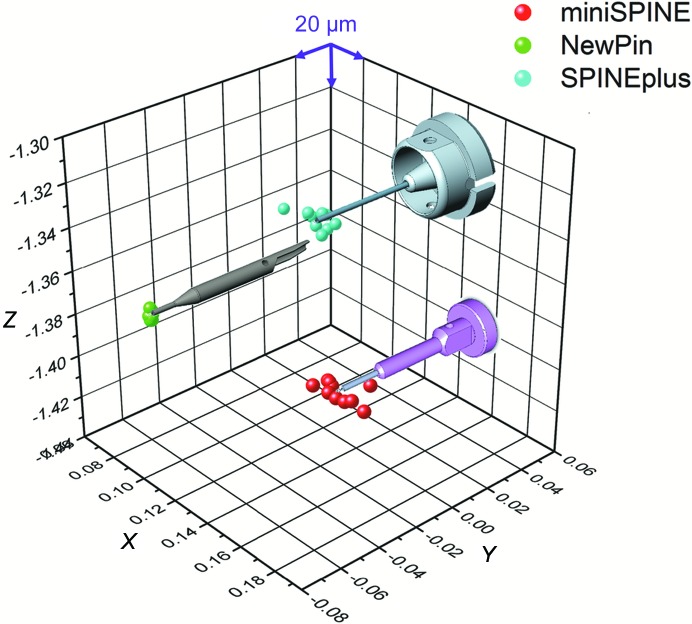
Sample-repositioning precision on a goniometer. The recorded centring positions for ten successive mounts for each sample standard are plotted in three dimensions (with corresponding sample holder).

The repositioning precision of a crystal on a goniometer is defined here as the three-dimensional position distribution between successive transfers. To have a comparative measure of the precision attainable with the three different sample-holder models, the following experimental protocol was used: (i) all sample holders were mounted with MiTeGen M2-10 MicroMount needles and stored in their corresponding pucks, (ii) all pucks were loaded into the EdgeDewar filled with liquid nitrogen and (iii) ten successive mounting/unmounting cycles were executed. After each mounting, the centre of the 10 µm MicroMount loop was manually centred to the beam position using the three-click centring feature of the MD2S goniometer (precision of ∼2 µm in three dimensions). The *X* and *Y* coordinates of the goniometer centring table, as well as the *Y* coordinate of the goniometer translation stage (pin-length correction), were recorded and the differences between successive cycles used to calculate the three-dimensional repositioning precision of the 10 µm MicroMount loop. The NewPin sample holder shows a maximum repositioning error of ±3.5 µm, while the miniSPINE and SPINE sample holders have maximum repositioning errors of ±19.2 and ±8.3 µm, respectively (Fig. 8 ▸). As expected, the best repositioning precision is obtained with NewPin. This value mainly depends on the manufacturing quality of the pins and the precision of the goniometer mount (Papp *et al.*, 2017 ▸). The results obtained with the miniSPINE and SPINE sample holders are more difficult to interpret as they mostly depend on the precision of the transfer robotics and the stiffness of the grippers. More experiments will be necessary to assess the differences that are observed. The maximum *X*/*Y* repositioning error of the SPINE and SPINEplus sample holders is limited to 140 µm, fixed by the mechanical play between the inner diameter of the pins and the outer diameter of the SmartMagnetP and their respective manufacturing tolerances [drawings SPINE Cap DM 16106A and Goniometer Mount DM 16300 available as Supporting Information to Cipriani *et al.* (2006 ▸); https://doi.org/10.1107/S0907444906030587/gx5085sup1.pdf]. There is no such limitation for the miniSPINE sample holder, which is mounted on the flat part of the SmartMagnetP. The design of a goniometer-mount specific to miniSPINE that provides improved *X*/*Y* sample positioning could be envisaged when compatibility with SPINE is not required.

## 4. Discussion   

A fast, flexible and reliable sample-changer family based on an industrial six-axis robot has been developed. The FlexED8 model described in this article allows a single beamline to accept both the popular SPINE sample holder and the newly developed high-density miniSPINE as well as NewPin sample holders. A sample-storage capacity of 252 samples is achieved with seven pucks stored in an open, 40 cm diameter, self-cleaning dewar (EdgeDewar ED8). Overall, the FlexED8 offers a compact, easily accessible, ice-free, high-capacity cryogenic sample-storage system. The nominal sample-exchange time of 40 s is reduced to less than 5 s for SPINE sample holders when a double gripper is used (Supplementary Fig. S2), and the development of a double gripper for the NewPin and miniSPINE sample holders is ongoing. The FlexED8 sample changer has been successfully tested on the EMBL–ESRF–India beamline BM14. Crystals were reliably transferred from the EdgeDewar to the goniometer without ice contamination and maintained at a temperature below 100 K throughout the loading and unloading procedures with a comfortable safety margin. The repositioning precision of ±3.5 µm obtained with the NewPin sample holders through successive loading and unloading cycles should decrease the duration of crystal alignment at beamlines and make it potentially unnecessary for crystals harvested by automated systems that provide the coordinates of the crystals (Svensson *et al.*, 2015 ▸; Zander *et al.*, 2016 ▸). The Flex sample-changer family is equipped with a tool-changer system that allows an expansion of the handling capabilities. An example is a gripper for SBS crystallization plates (Supplementary Fig. S6) currently under development that will be used in two different environments. Firstly, on ESRF beamline ID30B it will be deployed to automatically mount SBS crystallization plates stored in a hotel onto the plate holder of the MD2S gonio­meter for *in situ* data collection (Supplementary Fig. S7; FlexHCD). Secondly, it will be used at the crystal-harvesting station of the high-throughput crystallization laboratory of the EMBL Grenoble Outstation to transfer crystallization plates from the imaging systems (Formulatrix Rock Imager 1000) to the CrystalDirect harvester (Supplementary Fig. S7; FlexED3).

The emergence of X-ray free-electron lasers has led to the development of many new sample-delivery methods such as crystal jets (Weierstall *et al.*, 2014 ▸; Sugahara *et al.*, 2015 ▸) and micro-patterned chips (Oghbaey *et al.*, 2016 ▸; Lyubimov *et al.*, 2015 ▸; Coquelle *et al.*, 2015 ▸; Cohen *et al.*, 2014 ▸; Mueller *et al.*, 2015 ▸) and has promoted serial crystallography methods. More recently, with upgrade programs ongoing or planned at many synchrotrons worldwide (Eriksson *et al.*, 2014 ▸), data-collection times are foreseen to be dramatically reduced at MX beamlines. An increasing variety of crystal supports and sample-delivery methods is therefore anticipated for the next decade, making the paradigm of one crystal per mount obsolete. For these reasons, the future requirements at synchrotrons are difficult to anticipate and the flexibility of sample-delivery systems is an important consideration.

The Flex sample-changer family should be able to cope with the changing needs in automation at beamlines and increase the efficiency of the entire MX pipeline. The most recent example is the installation of a CrystalDirect harvester next to the FlexHCD sample changer at ESRF beamline ID30B. In this setup, the harvested crystals are directly mounted on the goniometer by the Flex robotics, and this new harvest-and-collect pipeline further streamlines the structure-solution process.

## Supplementary Material

Supplementary Figures. DOI: 10.1107/S2059798317013596/gm5054sup1.pdf

Video of loading-unloading sequence with miniSPINE sample holders.. DOI: 10.1107/S2059798317013596/gm5054sup2.wmv

Video of loading-unloading sequence with NewPin sample holders.. DOI: 10.1107/S2059798317013596/gm5054sup3.wmv

Video of loading-unloading sequence with SPINE sample holders, simple gripper.. DOI: 10.1107/S2059798317013596/gm5054sup4.wmv

Video of loading-unloading sequence with SPINE sample holders, double gripper.. DOI: 10.1107/S2059798317013596/gm5054sup5.wmv
