# Supplementary material for: FlexED8: the first member of a fast and flexible sample-changer family for macromolecular crystallography
Source: Acta Crystallogr D Struct Biol. 2017 Sep 29;73(Pt 10):841–51. doi: 10.1107/S2059798317013596 (PMC5633909; doi:10.1107/S2059798317013596)
Supplement: Supplementary file 1 [file d-73-00841-sup1.pdf]

STRUCTURAL
BIOLOGY

**Volume 73 (2017)**

**Supporting information for article:**

# FlexED8: the first member of a fast and flexible sample changer family for macromolecular crystallography

**Gergely Papp, Franck Felisaz, Clement Sorez, Marcos Lopez-Marrero, Robert Janocha, Babu Manjasetty, Alexandre Gobbo, Hassan Belrhali, Matthew W. Bowler and Florent Cipriani**

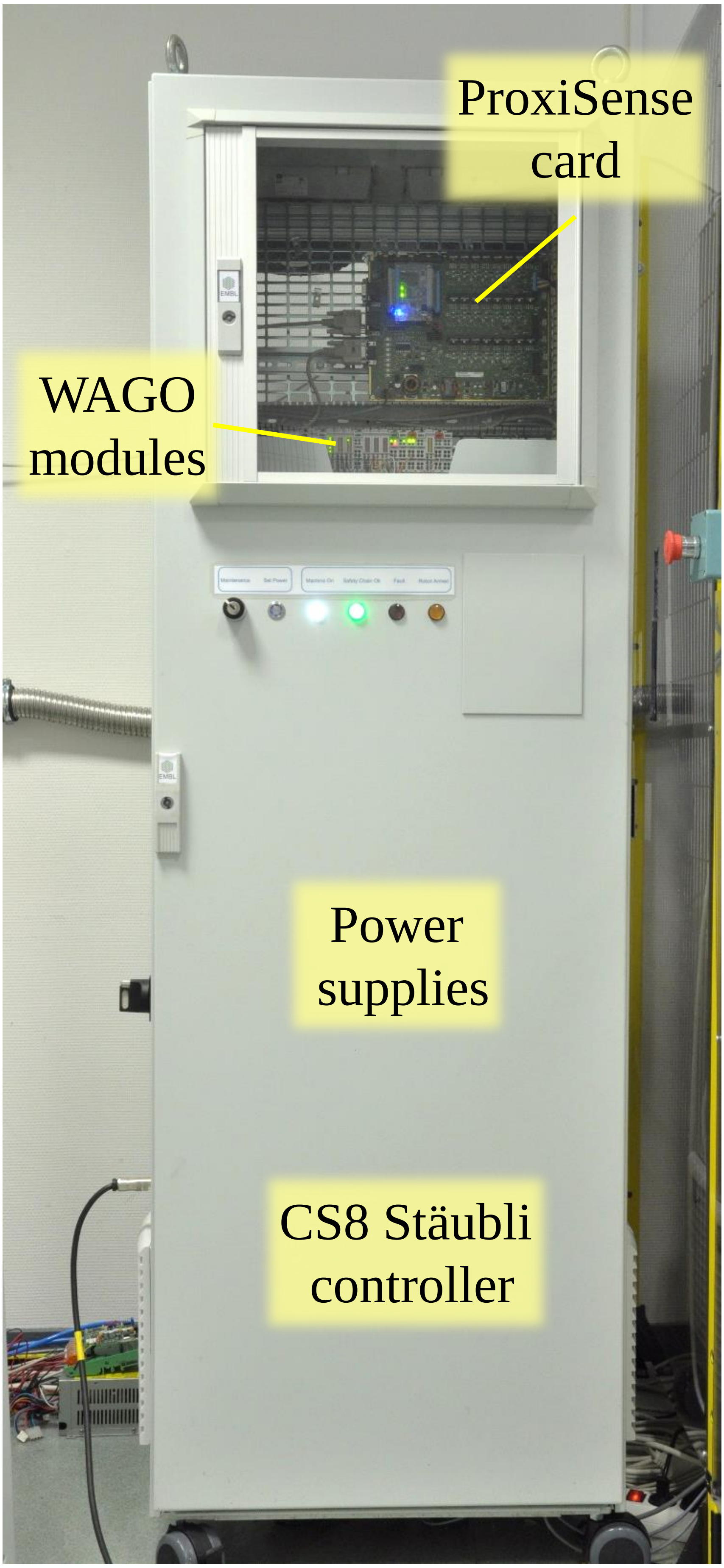


Figure S1. FlexED8 control cabinet that contains, visible though the front window, the ProxiSense card and the WAGO modules , and underneath the power supplies and CS8 Stäubli controller

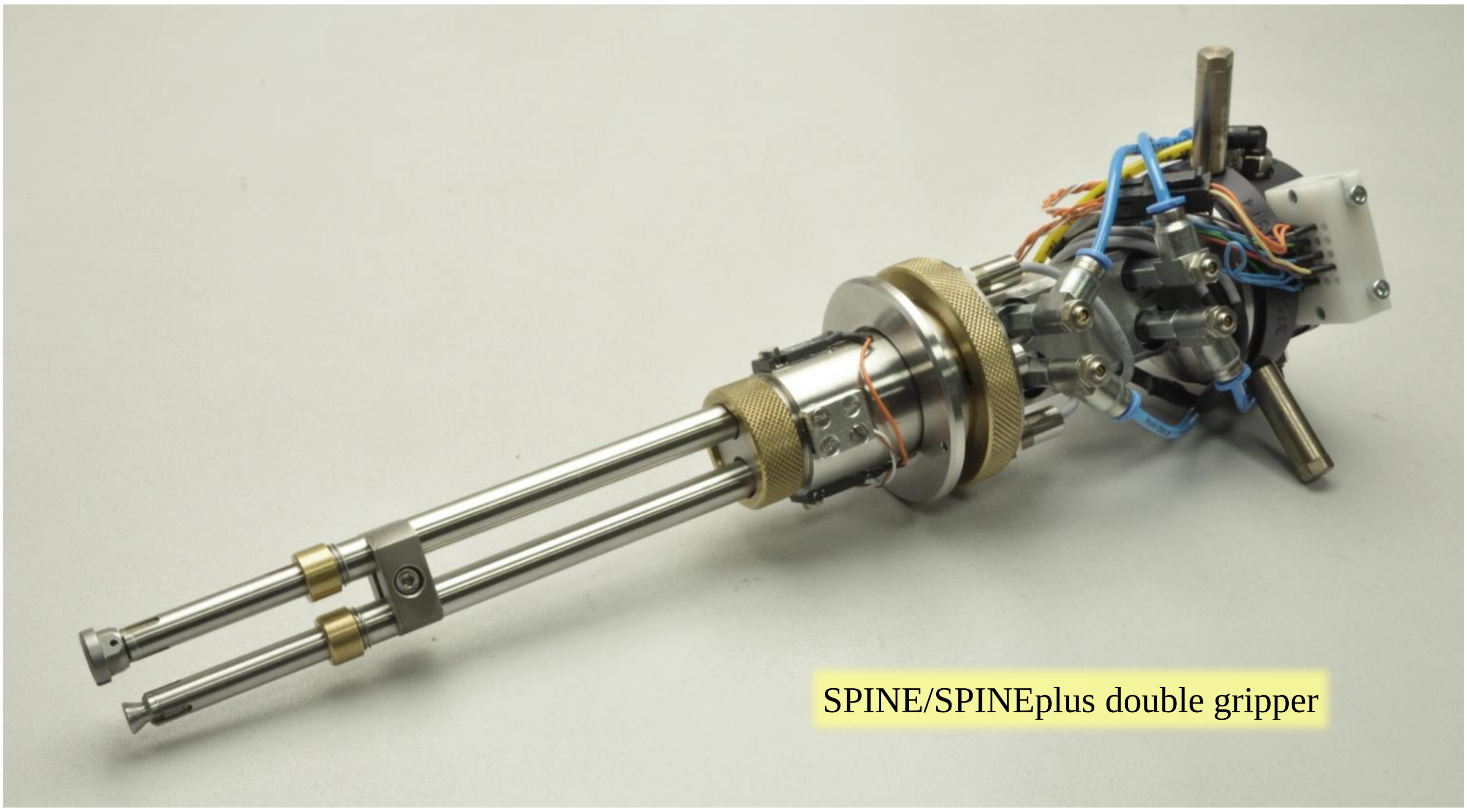


Figure S2. SPINE/SPINEplus double gripper

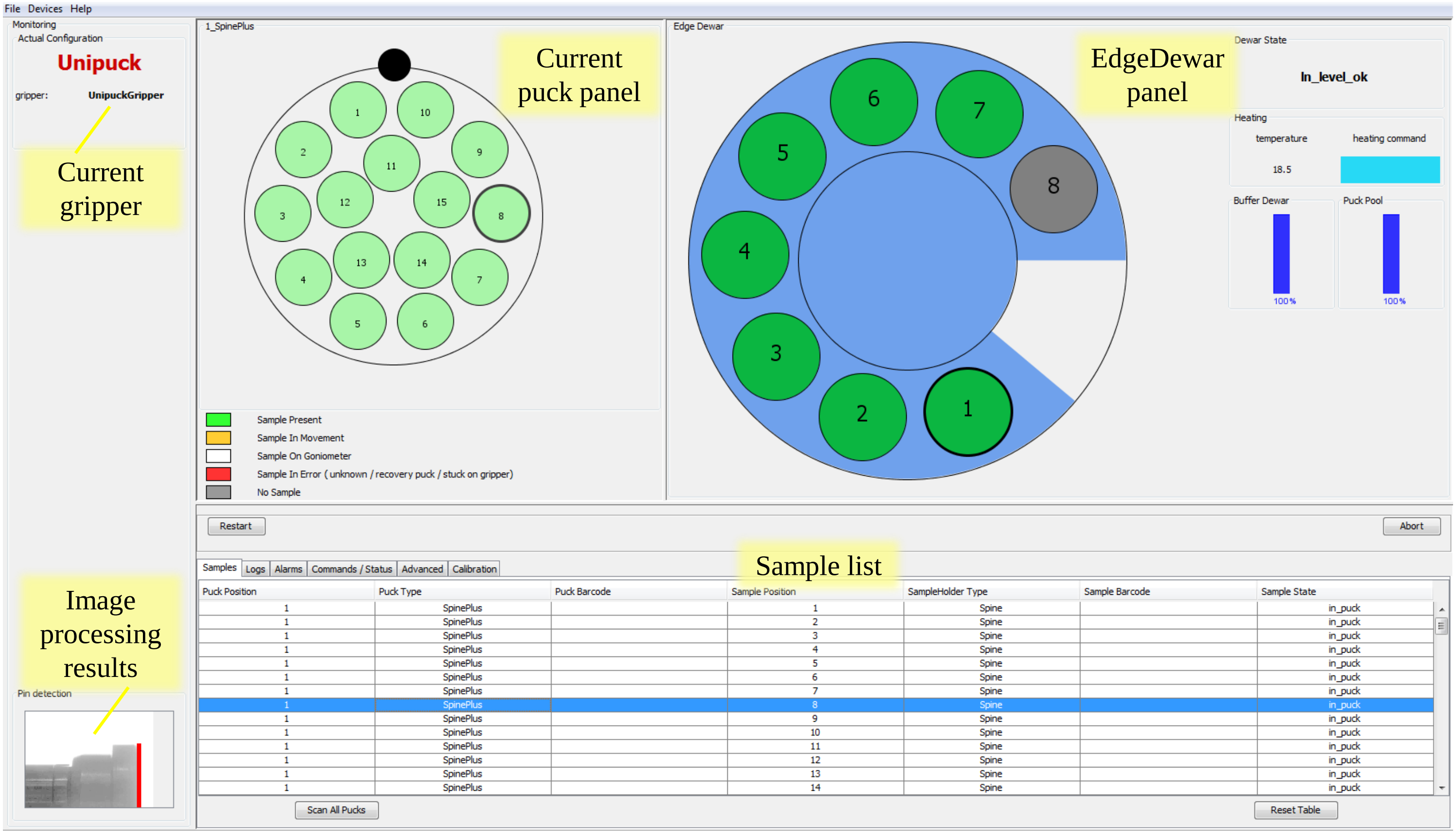


Figure S3. FlexED8 Graphical user interface: Sample transfer mode with 7 pucks loaded in the Dewar.

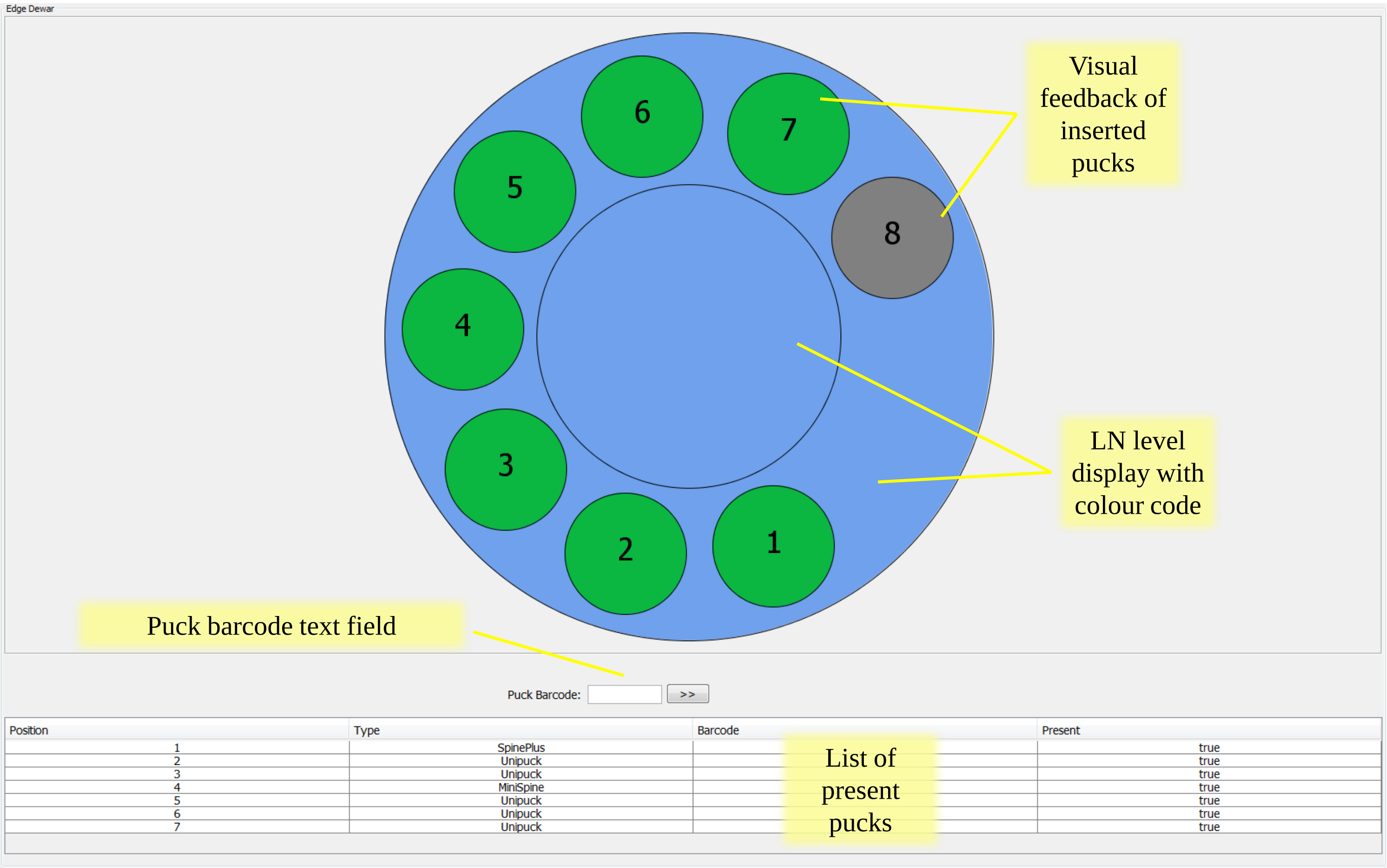


Figure S4. FlexED8 Graphical user interface: Dewar loading mode with 7 pucks loaded in the Dewar

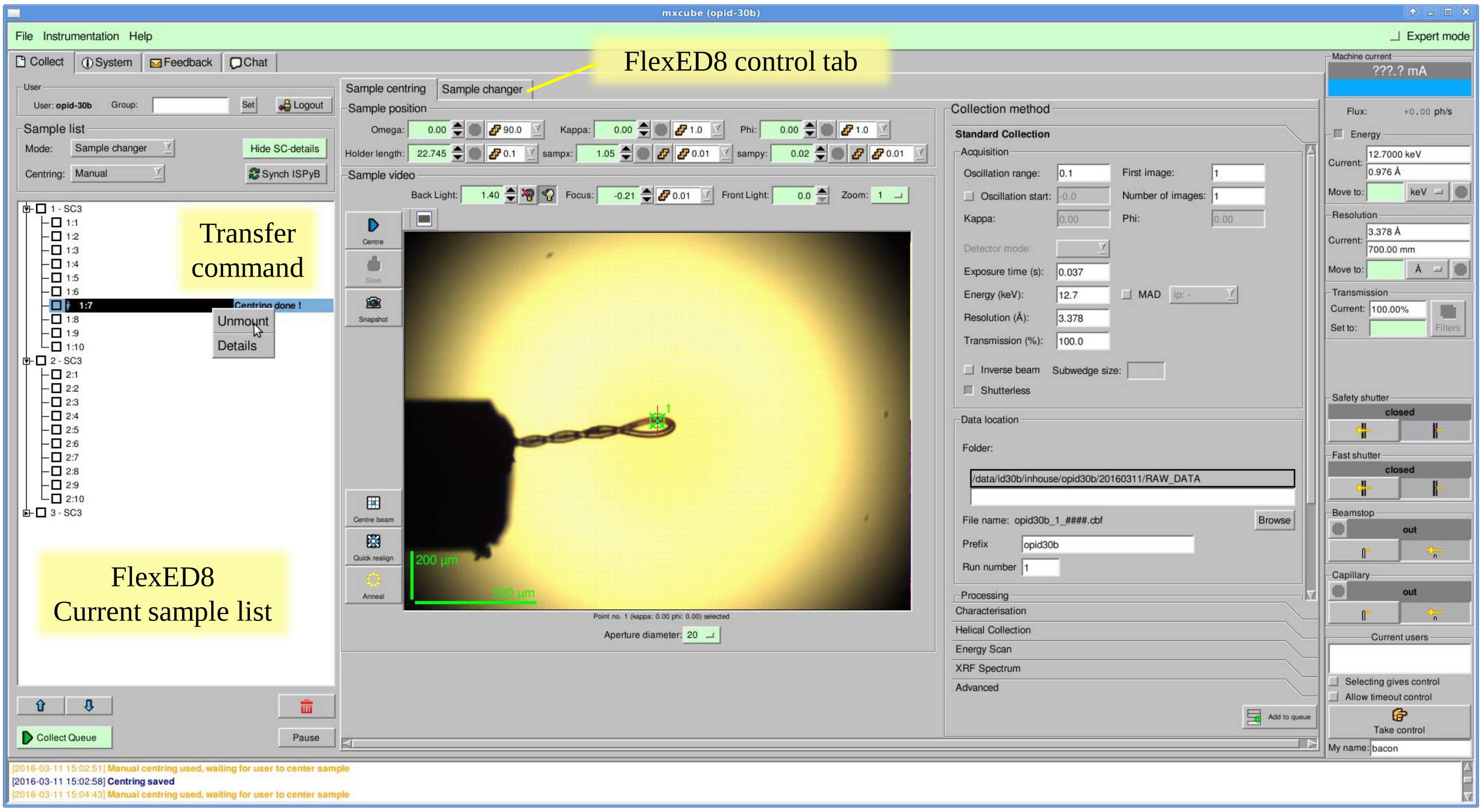


Figure S5. MXCuBE graphical user interface: Data collection tab, with on the left the generic sample changer control pane that directly controls the FlexED8 sample changer

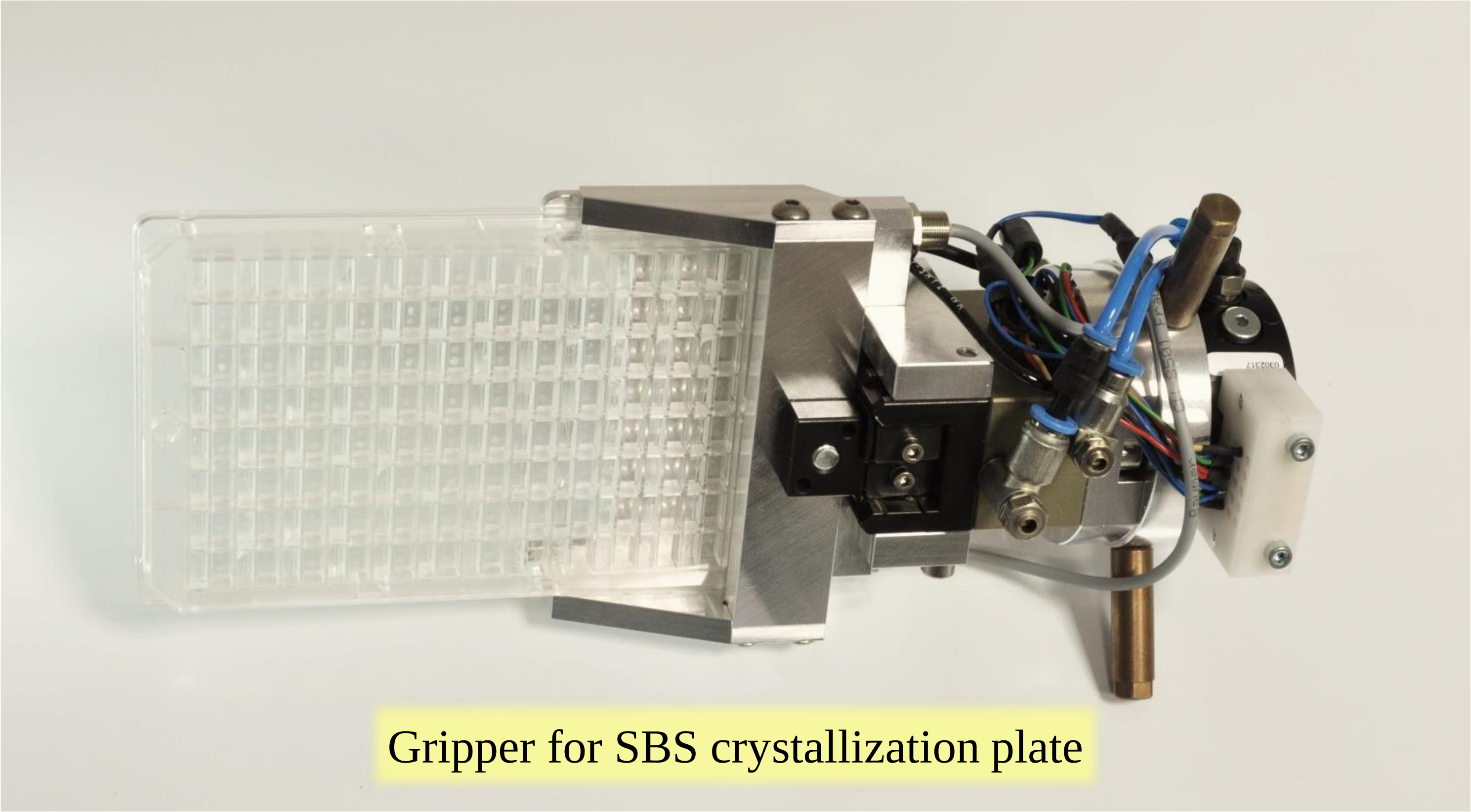


Figure S6.  Gripper for SBS crystallization plate

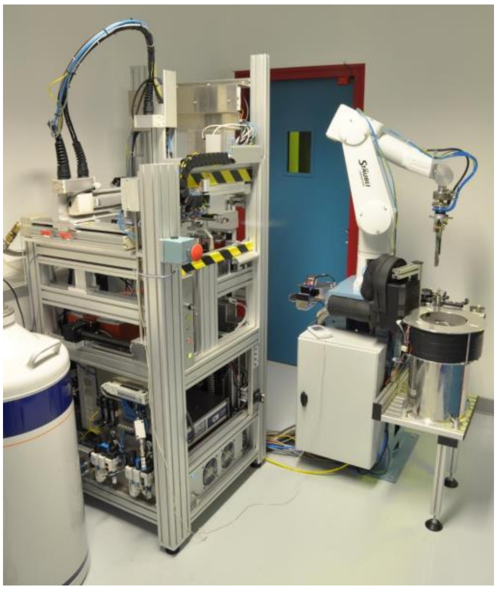

FlexED3
Cryo-storage unit

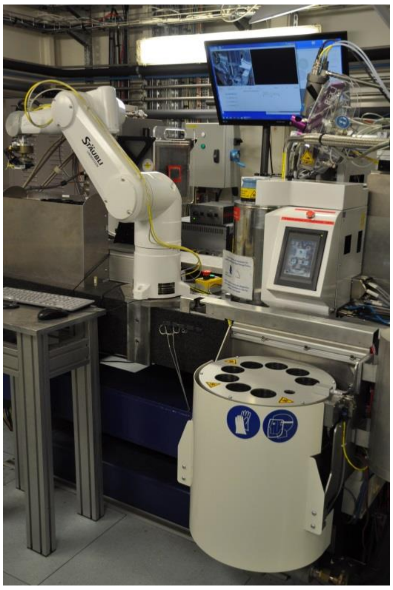

FlexED8
sample changer

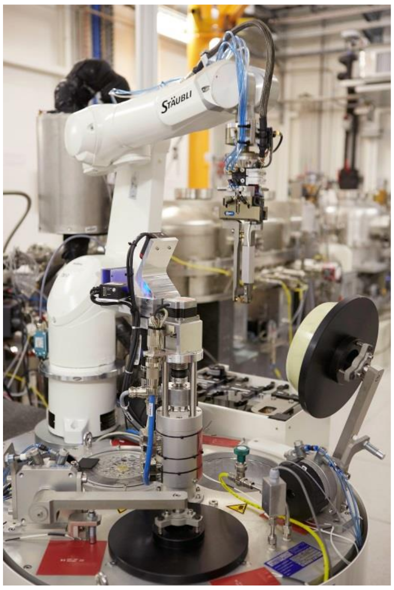

FlexHCD
Sample Changer

Figure S7. Flex robotics family members

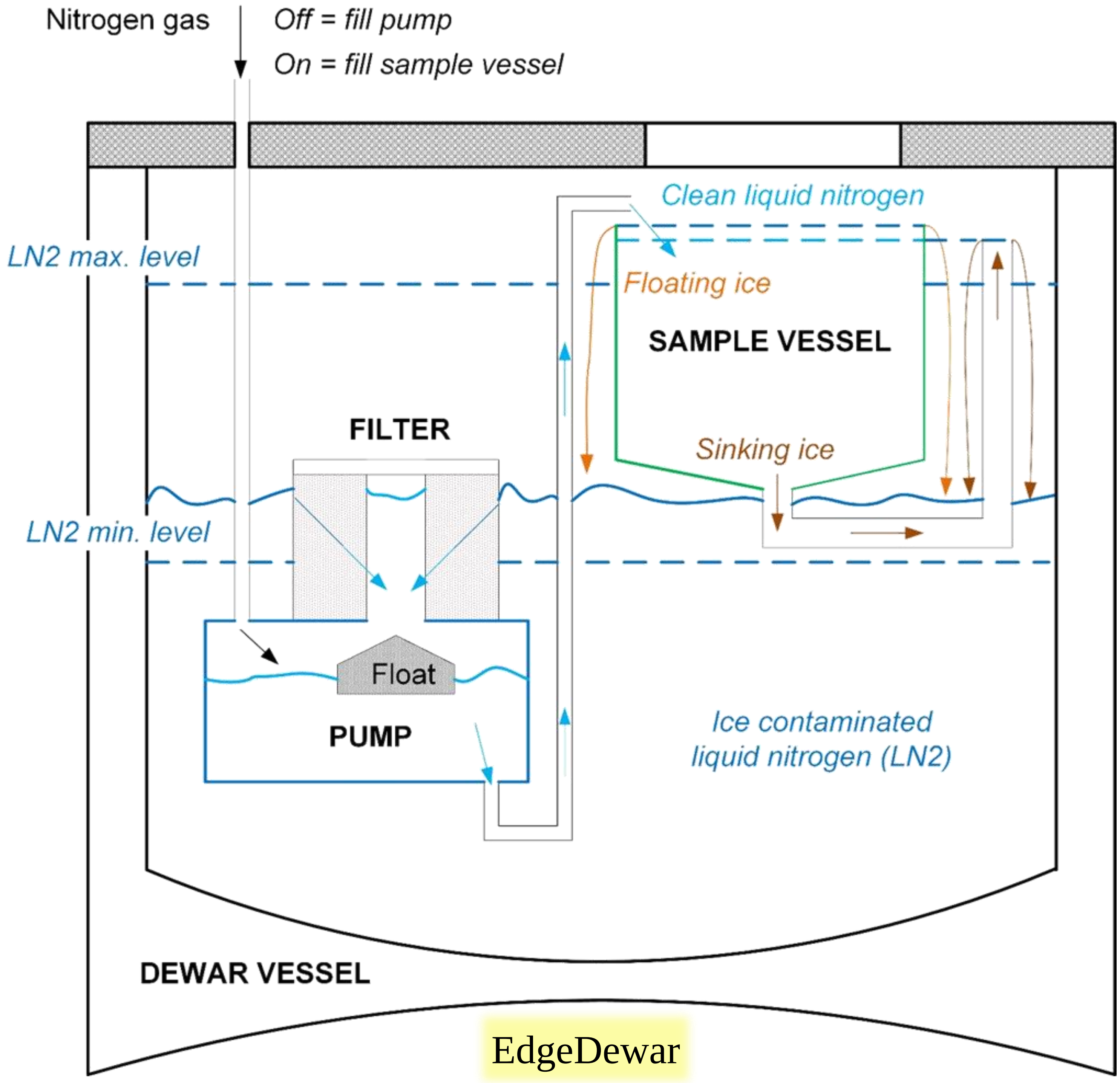


Figure S8. Principle of the EdgeDewar
